# Compounding and stability studies of liquid oral formulations of beta-blockers (bisoprolol, betaxolol, and nadolol) for paediatric patients

**DOI:** 10.3389/jpps.2025.15387

**Published:** 2025-12-02

**Authors:** Laura Dubois, Cyrielle Bouguergour, Romain Paoli-Lombardo, Caroline Castera-Ducros, Christophe Jean, Mélanie Fuchs, Patrice Vanelle, Pascal Rathelot, Thierry Terme, Christophe Curti

**Affiliations:** 1 Service Central de la Qualité et de l’information Pharmaceutiques (SCQIP), Assistance Publique – Hôpitaux de Marseille (AP-HM), Marseille, France; 2 Pharmacie Usage Intérieur Hôpital Nord, Assistance Publique - Hôpitaux de Marseille (AP-HM), Hôpital Nord, Marseille, France; 3 Aix-Marseille Université, Centre National de la Recherche Scientifique (CNRS), Institut de Chimie Radicalaire ICR, UMR 7273, Équipe de Pharmaco-Chimie Radicalaire, Marseille, France; 4 Pharmacie Usage Intérieur Hôpital Sainte-Marguerite, Assistance Publique - Hôpitaux de Marseille (AP-HM), Hôpital Sainte-Marguerite, Marseille, France; 5 Pharmacie Usage Intérieur Hôpital de la Timone, Assistance Publique - Hôpitaux de Marseille (AP-HM), Hôpital de la Timone, Marseille, France

**Keywords:** bisoprolol, betaxolol, nadolol, compounding, stability study

## Abstract

In paediatric wards, bisoprolol, betaxolol, or nadolol can be administered orally at non-licensed dosages. To provide paediatric patients with appropriate treatment, batches of beta-blocker oral suspensions must be compounded, which involves subsequent stability studies. A stability-indicating HPLC-UV method and microbiological analyses were validated. Experimental batches were compounded (betaxolol hydrochloride 1 mg.mL^−1^, bisoprolol hemifumarate 0.5 mg.mL^−1^ and nadolol 10 mg.mL^−1^). Bisoprolol hemifumarate 0.5 mg.mL^−1^ and nadolol 10 mg.mL^−1^ needed the addition of citric acid (3 mg.mL^−1^) and potassium sorbate (3 mg.mL^−1^) to ensure preservative efficacy. Betaxolol hydrochloride 1 mg.mL^−1^ was stable for 2 months when stored at 2–8  °C, for 1 month after opening at 2–8 °C, and for 15 days when stored at 25 °C/60% RH. Bisoprolol hemifumarate 0.5 mg.mL^−1^ was stable for 2 months when stored at 2–8 °C, for 1 month after opening at 2–8 °C, and for 15 days when stored at 25 °C/60% RH. Nadolol 10 mg.mL^−1^ was stable for 3 months when stored at 2–8 °C, for 1 month after opening at 2–8 °C, and for 15 days when stored at 25 °C/60% RH. Hospital pharmacies can compound batches of beta-blocker liquid oral suspensions and store them for secure dispensing and administration.

## Introduction

Several beta-blockers can be prescribed in paediatric wards when no commercial drugs are available. Betaxolol hydrochloride, administered orally at a dosage of 0.25 mg.kg^−1^, is used for the diagnosis of paediatric growth hormone deficiency (provocative growth hormone testing) [1, 2]. Bisoprolol hemifumarate is prescribed *per os* for various paediatric cardiac pathologies, such as paediatric heart failure [3, 4], congenital structural heart disease, or cardiomyopathy, with daily dosages ranging from 0.02 mg.kg^−1^ to 0.4 mg.kg^−1^ [5]. Nadolol is used in paediatric patients to treat ventricular arrhythmia at a dosage of 0.5–1 mg.kg^−1^ [6].

When a commercial preparation is not available, pharmacists must produce a medication. These preparations can be made extemporaneously for one patient. This method allows dosage individualisation, but is time-consuming and can lead to iatrogenic events, as quality controls are often not possible. Alternatively, preparations can be compounded in advance in batches and stored for dispensing to several patients [7–9]. This second possibility requires dosage harmonisation, costly stability studies, or, at least, a strong level of evidence to determine a beyond-use date. However, it is also more secure as batches can be controlled against pharmacopoeia specifications [10]. Moreover, as one batch is dispensed to several patients, the number of human resources involved in compounding is reduced. In our hospital, pharmacists have chosen, whenever possible, to prepare stored batches [11–14] to secure the process and to decrease costs. Therefore, paediatric beta-blockers are prescribed and extemporaneously compounded as oral liquid formulations in Syrspend® pH 4 (Fagron): betaxolol hydrochloride 1 mg.mL^−1^, bisoprolol hemifumarate 0.5 mg.mL^−1^ and nadolol 10 mg.mL^−1^.

During compounding, we discovered that nadolol from the raw material or bisoprolol from the commercial tablets alone in Syrspend® did not ensure preservative efficacy in the resulting suspensions. Therefore, citric acid (3 mg.mL^-1^) and potassium sorbate (3 mg.mL^−1^) were added. Stability-indicating methods were developed for betaxolol, bisoprolol, and nadolol, and stability studies were performed. Herein, we present the results of our investigations.

## Materials and methods

### Compounding of beta-blocker oral suspensions

Compounding of three experimental batches of 20 betaxolol hydrochloride 1 mg.mL^−1^ oral suspensions was performed as follows: a total of 25 20 mg commercial betaxolol hydrochloride tablets were transferred to a mortar and crushed with a pestle. The resulting powder was sieved (with a 200 µm test sieve, ISO 3310-1) and poured again into the mortar. A small quantity of Syrspend® liquid pH 4 (approximately 50 mL) was added, and the resulting mixture was gently poured and homogenised with a pestle to obtain a paste. Syrspend® was then added q.s. (*Quantum satis*) to a final volume of 250 mL under continuous mixing, after which the suspension was poured into a beaker. The remaining Syrspend was added under manual stirring for 10 min to obtain a final volume of 500 mL. Finally, the suspension was equally divided with a 60 mL syringe into 30 mL brown glass bottles, with each bottle receiving 25 mL of the mixture. The bottles were sealed with screw caps and labelled. The same procedure was used to obtain 3 experimental batches of 20 bisoprolol hemifumarate 0.5 mg.mL^−1^ oral suspensions, replacing betaxolol tablets with 25 bisoprolol 10 mg tablets and adding 1,500 mg of citric acid (Cooper, Paris, France) and 1,500 mg of potassium sorbate (Fagron, Thiais, France). Finally, to obtain three experimental batches of 20 nadolol 10 mg.mL^−1^ oral suspensions, 5,000 mg of nadolol raw material were used, along with 1,500 mg of citric acid and 1,500 mg of potassium sorbate (Fagron, Thiais, France).

### HPLC-UV methods

Method validation and forced degradation studies were performed with betaxolol hydrochloride analytical standards (reference (ref) 1069903 and ref B1103000, Sigma-Aldrich, Saint-Quentin-Fallavier, France), with bisoprolol hemifumarate analytical standards (ref 50787-25 MG, Sigma-Aldrich, Saint-Quentin-Fallavier, France), and with nadolol analytical standards (ref Y0000146 and ref PHR3212-400 MG, Sigma-Aldrich, Saint-Quentin-Fallavier, France). Betaxolol hydrochloride 20 mg tablets (Kerlone®, Cheplapharm, France), bisoprolol hemifumarate 10 mg tablets (BisoCe®, Mylan, France), and nadolol pharmaceutical-quality raw material (Inresa, France) were used for compounding.

The HPLC mobile phases were prepared using ultrapure water from a water purification system (Direct-Q® 3 UV, Merck, Lyon, France) and HPLC-grade methanol (HiPerSolv Chromanorm®, VWR International, Rosny-sous-bois, France). The other chemical products used were potassium dihydrogen phosphate (VWR International, Rosny-sous-bois, France) and 85% orthophosphoric acid (Normapur®, VWR International, Rosny-sous-bois, France).

The bisoprolol and betaxolol mobile phases consisted of a mixture of an aqueous solution of 0.02 M potassium dihydrogen phosphate, adjusted to pH = 3 with orthophosphoric acid (45%, v/v) and methanol (55%, v/v). The nadolol mobile phase consisted of an aqueous solution of 0.02 M potassium dihydrogen phosphate adjusted to pH = 3 with orthophosphoric acid (75%, v/v) and methanol (25%, v/v). The mobile phases were filtered through Millipore 0.45 µm cellulose filters and used in isocratic mode at a flow rate of 1 mL/min for 15 min. The wavelength for beta-blocker detection was 220 nm, with an injection volume of 20 µL.

Volumes were aliquoted with a precision pipette (Thermo Scientific Finnpipette® F2 500 µL, Thermo Fisher Scientific, Illkirsch, France). Chromatographic analysis was carried out on an automated high-performance liquid chromatography Dionex Ultimate 3000® system equipped with a UV diode array detector. The apparatus was connected to an HP 1702 computer equipped with chromatographic data processing software (chromatographic data system Chromeleon® version 7.2.10, Thermo Fisher Scientific, Illkirsch, France). A C18 column (Stability®, 4.6 × 150 mm, 5 µm, ref 720014-46, Macherey-Nagel, Hoerdt, France) was used to separate the beta-blockers.

### Beta-blocker dosing method validation

First, stock solutions of the beta-blockers in ultrapure water were prepared: 500 μg.mL^−1^ for betaxolol hydrochloride and nadolol and 200 μg.mL^−1^ for bisoprolol (as hemifumarate).

The linearity of the HPLC-UV standard curve for beta-blockers was determined using five concentrations ranging from 25 to 200 μg.mL^−1^ for bisoprolol and seven dilutions from 25 to 500 μg.mL^−1^ for betaxolol hydrochloride and nadolol, respectively. The bisoprolol dosing method was found to be linear between 25 and 200 μg.mL^−1^, the nadolol dosing method was found to be linear between 25 and 500 μg.mL^−1^, whereas the betaxolol hydrochloride dosing method was found to be linear between 50 and 500 μg.mL^−1^ but not at the lowest dilution level of 25 μg.mL^−1^. Repeatability, intermediate precision, accuracy were determined at three concentration levels, and the relative standard deviation (RSD) was determined. The results are reported in Table 1.

**TABLE 1:** Repeatability, intermediate precision, and accuracy.

Samples	% RSD within-day	% RSD between-day	Accuracy (Bias in %)
Betaxolol hydrochloride 90 μg.mL^−1^	0.408%	0.672%	−0.247%
Betaxolol hydrochloride 100 μg.mL^−1^	0.296%	0.797%	−0.191%
Betaxolol hydrochloride 110 μg.mL^−1^	0.449%	0.721%	−0.528%
Bisoprolol 90 μg.mL^−1^ (as hemifumarate)	2.557%	1.562%	0.946%
Bisoprolol 100 μg.mL^−1^ (as hemifumarate)	1.002%	1.129%	0.837%
Bisoprolol 110 μg.mL^−1^ (as hemifumarate)	0.961%	2.268%	−0.090%
Nadolol 180 μg.mL^−1^	0.123%	0.829%	−0.079%
Nadolol 200 μg.mL^−1^	0.991%	0.514%	0.504%
Nadolol 220 μg.mL^−1^	0.204%	0.956%	−0.307%

**FIGURE 1:**
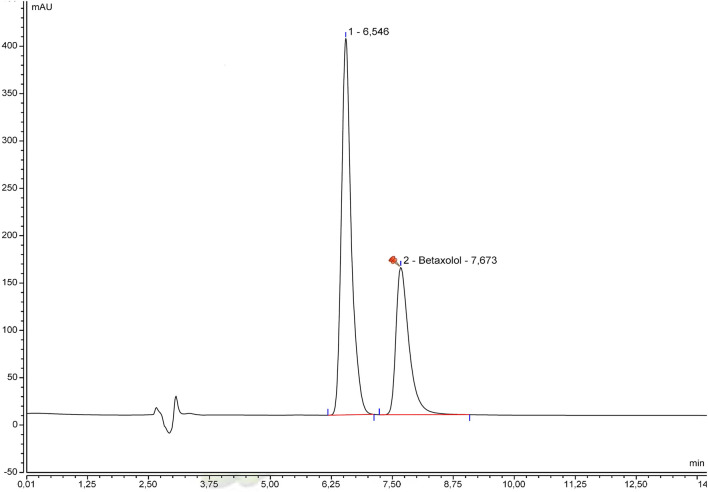
Representative betaxolol hydrochloride suspension chromatogram.

**FIGURE 2:**
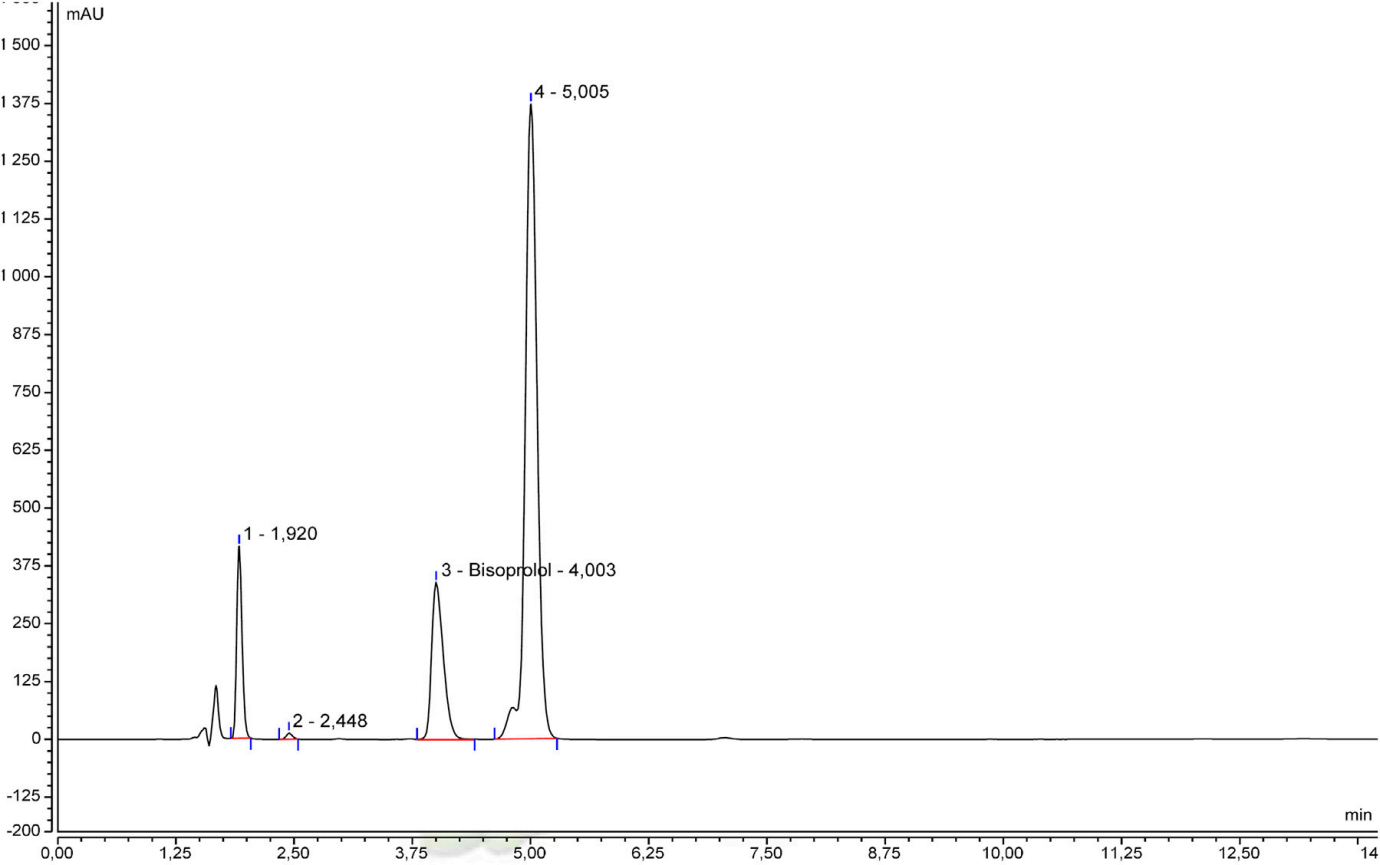
Representative bisoprolol hemifumarate suspension chromatogram.

**FIGURE 3:**
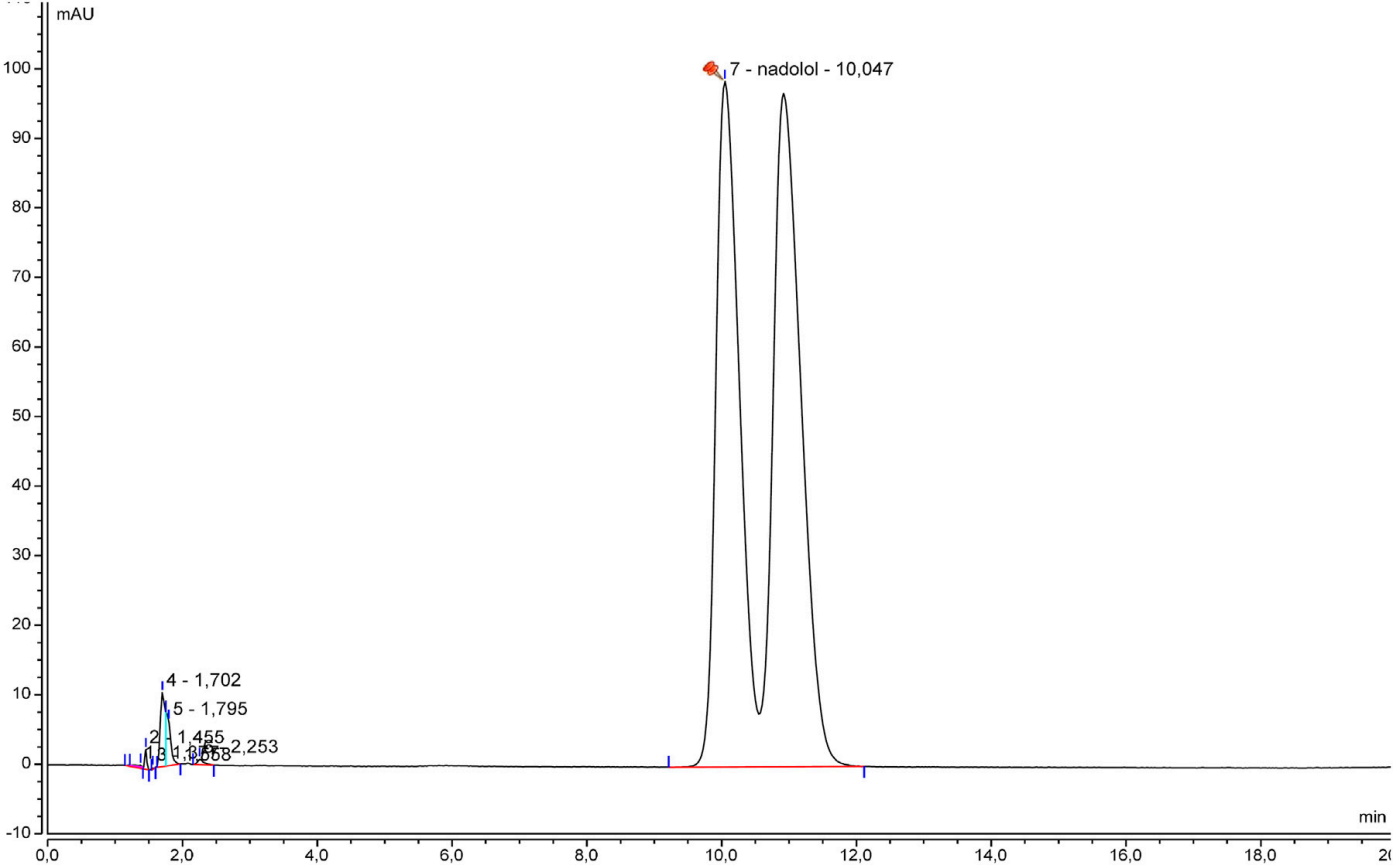
Representative nadolol suspension chromatogram.

The specificity was initially examined by comparing the beta-blocker alone and with excipients. Chromatograms of betaxolol hydrochloride (Figure 1), bisoprolol hemifumarate (Figure 2), and nadolol (Figure 3) oral suspensions showed no interference due to excipients.

Then, specificity was studied through forced degradation studies.

Stock solutions of betaxolol hydrochloride (500 μg.mL^−1^), bisoprolol (200 μg.mL^−1^), and nadolol (500 μg.mL^−1^) were diluted and exposed to several stress conditions, including intense heating, oxidation, exposure to light (20 cm from a 20W compact fluorescent lamp with a colour temperature of 2700 K), and extreme pH values. Theoretical concentrations obtained after dilution with ultrapure water or with reactant (aqueous HCl, NaOH, or H_2_O_2_) were 100 μg.mL^−1^ for betaxolol hydrochloride and bisoprolol and 200 μg.mL^−1^ for nadolol, respectively. The beta-blocker content after each degradation was compared with that of a freshly made standard to determine the percentage of degradation. Degradation products were identified and reported with their Relative Retention Time (RRT), as compared with the beta-blocker peak. The results are summarised in Table 2. Representative chromatograms for each condition are represented in the Supplementary Material.

**TABLE 2:** Forced degradation of beta-blockers.

Sample	Experimental condition	% degrad	Degradation products (RRT)
Betaxolol	Light (artificial light, 7 days)	8%	0.51; 0.57; 0.80; 1.70
	Heat (80 °C, 5 days)	6%	0.51; 0.80; 1.70
	Acidic (HCl, 2.5M, 15 min)	28%	0.42; 0.58; 0.70; 0.75
	Alkaline (NaOH 0.1 M, 30 min)	12%	0.38
	Oxidation (H_2_O_2_ 3%, 2 h)	28%	0.51; 0.61; 1.19
Bisoprolol	Light (artificial light, 2 days)	3%	1.27; 1.40; 1.88
	Heat (50 °C, 2 days)	44%	0.65
	Acidic (HCl 2M, 1 h)	4%	1.40
	Alkaline (NaOH 0.1 M, 15 min)	4%	0.39; 0.65; 1.40; 1.88
	Oxidation (H_2_O_2_ 3%, 15 min)	2%	0.49; 1.55
Nadolol	Light (artificial light, 14 days)	9%	0.79
	Heat (80 °C, 14 days)	12%	0.36; 0.40; 0.54; 0.70; 0.79; 2.17
	Acidic (HCl 2.5 M, 14 days)	4%	1.53
	Alkaline (NaOH 4 M, 6 days)	2%	0.57
	Oxidation (H_2_O_2_ 15%, 5 h)	3%	0.20; 0.27; 0.32; 0.70; 0.86; 1.33; 3.84

During each forced degradation test, no interference between each beta-blocker and its degradation products was observed.

Finally, to ensure robustness, System Suitability Tests (SSTs) were defined for each beta-blocker, based on the relevant USP, monograph or, if unavailable, the FDA, recommendation for pharmaceutical analysis [15]. The values are reported in Table 3.

**TABLE 3:** System Suitability Tests for beta-blockers: stability-indicating dosing methods.

Parameter	Betaxolol hydrochloride	Bisoprolol (as hemifumarate)	Nadolol
K’ (capacity factor)	>2	>2	>2
Rs (resolution)	>2	>7^a^	>2
N (theoretical plates)	>2,000	>2,000	>2,000
T (tailing factor)	<3^b^	≤2^a^	≤3^c^
RSD (from six analyses)	1%^b^	2%^a^	2%^c^

^a^ Bisoprolol fumarate tablets monograph, USP.

^b^ Betaxolol tablets monograph, USP.

^c^ Nadolol tablets monograph, USP.

The betaxolol hydrochloride 1 mg.mL^−1^ oral suspension was treated as follows: 2.5 mL were solubilised in 17.5 mL of ultrapure water, vortexed for 1 min, and centrifuged for 5 min at 448 G (2,000 RPM with Megafuge 16 Heraeus®, Thermo Scientific). A theoretical 100 μg.mL^−1^ betaxolol hydrochloride solution was obtained.

Bisoprolol hemifumarate 0.5 mg.mL^−1^ oral suspension was treated as follows: 0.59 mL was solubilised in 1.91 mL of mobile phase, vortexed for 1 min, and centrifuged for 5 min at 448 G. A theoretical 118 μg.mL^−1^ bisoprolol hemifumarate solution, equivalent to 100 μg.mL^−1^ of bisoprolol, was obtained.

Nadolol 10 mg.mL^−1^ oral suspension was treated as follows: 0.5 mL was solubilised in 24.5 mL of ultrapure water, vortexed for 1 min, and centrifuged for 5 min at 448 G. A theoretical 200 μg.mL^−1^ nadolol solution was obtained.

### Microbiological methods

Microbiological analyses of betaxolol hydrochloride 1 mg.mL^−1^, bisoprolol hemifumarate 0.5 mg.mL^−1^ and nadolol 10 mg.mL^−1^ suspensions were performed with a microbiological safety cabinet (MSC) (Herasafe® KS, Thermo Fisher Scientific, Illkirsch, France) using a filtration method with a Microsart® apparatus (Sartorius, France). The suitability of the method was proved in accordance with the European Pharmacopoeia: first, a 5 mg.mL^−1^ of α-amylase solution was prepared as follows: 45 mg of α-amylase (ref A4551−1G, Sigma-Aldrich, Saint-Quentin-Fallavier, France) was weighed on a precision balance. Inside the microbiological safety cabinet, these were dissolved in 9 mL of European Pharmacopoeia diluent pH7 (VWR International, Rosny-sous-bois, France, ref AX011158) and aseptically filtered with a 0.2 µm Minisart® filter (Sartorius, Aubagne, France). The α-amylase solution reacts with Syrspend® starch to avoid membrane clogging during filtration. Five reference strains (Biomérieux, Marcy L’étoile, France, Bioball®: *Staphylococcus aureus* NCTC10788, *Bacillus subtilis* NCTC10400, *Pseudomonas aeruginosa* NCTC12924, *Candida albicans* NCPF3179, and *Aspergillus braziliensis* NCPF2275) were used by direct inoculation into Letheen Broth bottles (Clearline, Bernolsheim, France, ref 693402).

A total of 24 Letheen Broth 90 mL bottles were placed under the microbiological safety cabinet. In total, 14 were used for bacteriological validation: 2 with diluted beta-blocker suspension (2.5 mL of a 1:10 dilution with EP diluent pH 7) alone, 2 with *Staphylococcus* alone, 2 with *Staphylococcus* and 2.5 mL of diluted suspension, 2 with *Bacillus*, 2 with *Bacillus* and 2.5 mL of diluted suspension, 2 with *Pseudomonas*, and 2 with *Pseudomonas* and 2.5 mL of diluted suspension. In total, 10 Letheen Broth 90 mL bottles were used for mycological validation: 2 with 1 mL of pure beta-blocker suspension, 2 with *Candida*, 2 with *Candida* and 1 mL of suspension, 2 with *Aspergillus* and 2 with *Aspergillus*, and 1 mL of suspension. A total of 1 mL of the sterile 5 mg.mL^−1^ α-amylase solution was added to each of the 24 Letheen Broth bottles.

After manual homogenisation of the 24 bottles of Letheen Broth for 5 min, each bottle was filtered using a Microsart® apparatus and a Microsart® 100 filter (Sartorius, Aubagne, France, ref 16D03-10-H6-TG) under a microbiological safety cabinet. For bacteriological validation, 14 Tryptic Soy Agar (TSA) plates (Sartorius, Aubagne, France) were used, and 10 Sabouraud agar plates (Sartorius, Aubagne, France) were used for mycological validation.

The tryptic soy agar plates were incubated for 3 days in an incubator at 30–35 °C and the Sabouraud agar plates were incubated for 5 days at 20–25 °C (Heratherm® and Heraeus® Thermo Fisher Scientific, Illkirsch, France). At the end of the incubation period, for each reference strain, the mean CFU count of the “suspension + reference strain” samples did not differ by more than a factor of two from the mean CFU count of the “reference strain alone” samples.

This protocol was routinely applied to batch control. For total aerobic microbial count (TAMC) analysis, 2.5 mL of a 1:10 dilution with EP diluent pH7 of the beta-blocker suspension and 1 mL of a 5 mg.mL^−1^ α-amylase solution were placed in a 90 mL Letheen Broth bottle in duplicate. For total combined yeast and mould count (TYMC) analysis, 1 mL of the pure beta-blocker suspension and 1 mL of a 5 mg.mL^−1^ α-amylase solution were placed in a 90 mL Letheen Broth bottle in duplicate. Batch conformity was established if the TAMC value was less than 200 CFU/mL (i.e., the mean CFU count value on TSA was lower than 50) and if the TYMC value was less than 20 CFU/mL (i.e., the mean CFU count value on Sabouraud agar was lower than 20).

Preservative efficacy was studied using four reference strains (Biomérieux, Marcy L’étoile, France®): *S. aureus* NCTC10788 (Bioball®, 1.1 × 10^8^ CFU, ref 56148), *P. aeruginosa* NCTC12924 (Bioball®, 1.1 × 10^8^ CFU, ref 56147), *C. albicans* NCPF3179 (Bioball®, 1.1 × 10^8^ CFU, ref 56145), and *A. braziliensis* NCPF2275 (Bioball®, 1.1 × 10^8^ CFU, ref 410106). On day 0 (D0), four beta-blocker suspension samples were contaminated with 10^5^–10^6^ CFU/mL of each reference strain, and one beta-blocker suspension sample was used as a negative control. The evolution of the contamination was studied with TAMC and TYMC analyses at D0, D14 and D28. The logarithmic decrease in micro-organism count must be at least equal to 3 log_10_ units for bacterial strains and at least equal to one log_10_ unit for fungal strains by D14. On D28, these values cannot be higher than those reported on D14.

### Other analytical methods

Viscosity was evaluated with a Brookfield® viscosimeter (LV DV 1M EZ-Lock), which was equipped with a small sample adapter (allowing temperature regulation with a water flow) and calibrated with silicon viscosity standards (RT50 and RT500, Paragon Scientific, LGC Standard, Molsheim, France®). Analyses were performed at 50 rpm for betaxolol and at 20 rpm for bisoprolol and nadolol.

The pH was measured with a FiveEasy F20 pH meter (Mettler Toledo) and calibrated with buffer solutions traceable to NIST at pH 4 and pH 7 (Thermo Fisher Scientific, Illkirsch, France).

### Stability studies

Stability studies were performed on three independent batches under two different storage conditions (2–8 °C and 25 °C/60% RH), and the following parameters were evaluated at several sampling points: Active Pharmaceutical Ingredient (API) content and degradation products, pH, viscosity, microbiological counts (TAMC and TYMC) and preservative efficacy.

For stability studies performed on hospital formulations, a significant change in API content is generally defined as a change of +/− 0% from the initial value [16–20]. Conformity for pH values was established if the pH remained within +/− 0.5 pH units of the initial value. Viscosity was considered stable if the measured value was within +/− [10 + measurement uncertainty]%. Microbiological analyses (TAMC, TYMC, and preservative efficacy) had to be consistent with European Pharmacopoeia specifications [21, 22].

The limit values for unknown degradation products were determined with ICH Topic Q3B (R2) “Impurities in New Drug Products” [23]. The three beta-blockers studied had maximum daily doses ranging from more than 10 mg to less than 1 g. ICH Q3B defines a reporting threshold at 0.10%, an identification threshold at 0.20%, and a qualification threshold at 0.20% or 0.50% (depending on the maximum daily dose). Unlike unpublished stability studies carried out by pharmaceutical companies before marketing authorisation, qualification and often identification cannot be performed during small-scale stability studies of compounded drugs. Indeed, these studies do not benefit from the financial and technical support provided by a marketing authorisation procedure. Limit values for degradation products are always very difficult to establish and are often underevaluated (leading to a subsequent underestimation of the stability of compounded drugs). In our study, we considered that if any unknown degradation products appeared during the study and increased by more than 0.20% (the identification threshold), the compounded drug product was not considered to be stable, as we were unable to identify them. Moreover, at the beginning of the stability study, the impurity profile of the compounded drug was established (with the relative area of each unknown peak), and if any unknown degradation products initially present in the impurity profile increased by more than 0.20% from their initial value, the compounded drug product would also not be considered to be stable. This limitation certainly underestimates the stability of beta-blocker oral suspensions, but small-scale compounded preparations do not need a duration of stability as long as commercial drugs have, as they have smaller batch sizes.

## Results

The preservative efficacy was initially tested on betaxolol hydrochloride 1 mg.mL^−1^, bisoprolol hemifumarate 0.5 mg.mL^−1^, and nadolol 10 mg.mL^−1^ in Syrspend pH 4 without the addition of citric acid and potassium sorbate. Tests were performed during the stability study at D0, D30, D60, and D90 under refrigeration conditions and at D0 and D15 under ambient storage conditions. For betaxolol hydrochloride, the preservative efficacy was compliant for each strain tested throughout the whole stability study. For bisoprolol hemifumarate, the preservative efficacy failed for the *A. braziliensis* strain from the beginning of the stability study (D0) onwards and at each sampling point. The stability study was stopped, after which a second stability study was performed on bisoprolol hemifumarate 0.5 mg.mL^−1^/potassium sorbate 3 mg.mL^−1^/and citric acid 3 mg.mL^−1^, in which the preservative efficacy was compliant at each sampling point. For nadolol, preservative efficacy was initially compliant at D0 and D15 (under ambient storage conditions) but failed at D30 and D60 of the stability study, for both the *A. braziliensis* and *P. aeruginosa* strains. The stability study was interrupted. During the second stability study performed on a formulation containing nadolol 10 mg.mL^−1^/potassium sorbate 3 mg.mL^−1^/and citric acid 3 mg.mL^−1^, the preservative efficacy was compliant at each sampling point.

TAMC and TYMC remained compliant (TAMC <200 CFU.mL^−1^ and TYMC <20 CFU.mL^−1^) throughout the stability studies, for each storage condition of each beta-blocker. The pH value did not vary by more than +/- 0.5 pH units from its initial value for each storage condition of each beta-blocker. The viscosity value did not vary by more than +/− [10 + measurement uncertainty]% from its initial value for each storage condition of each beta-blocker, with one notable exception: bisoprolol hemifumarate.

**FIGURE 4:**
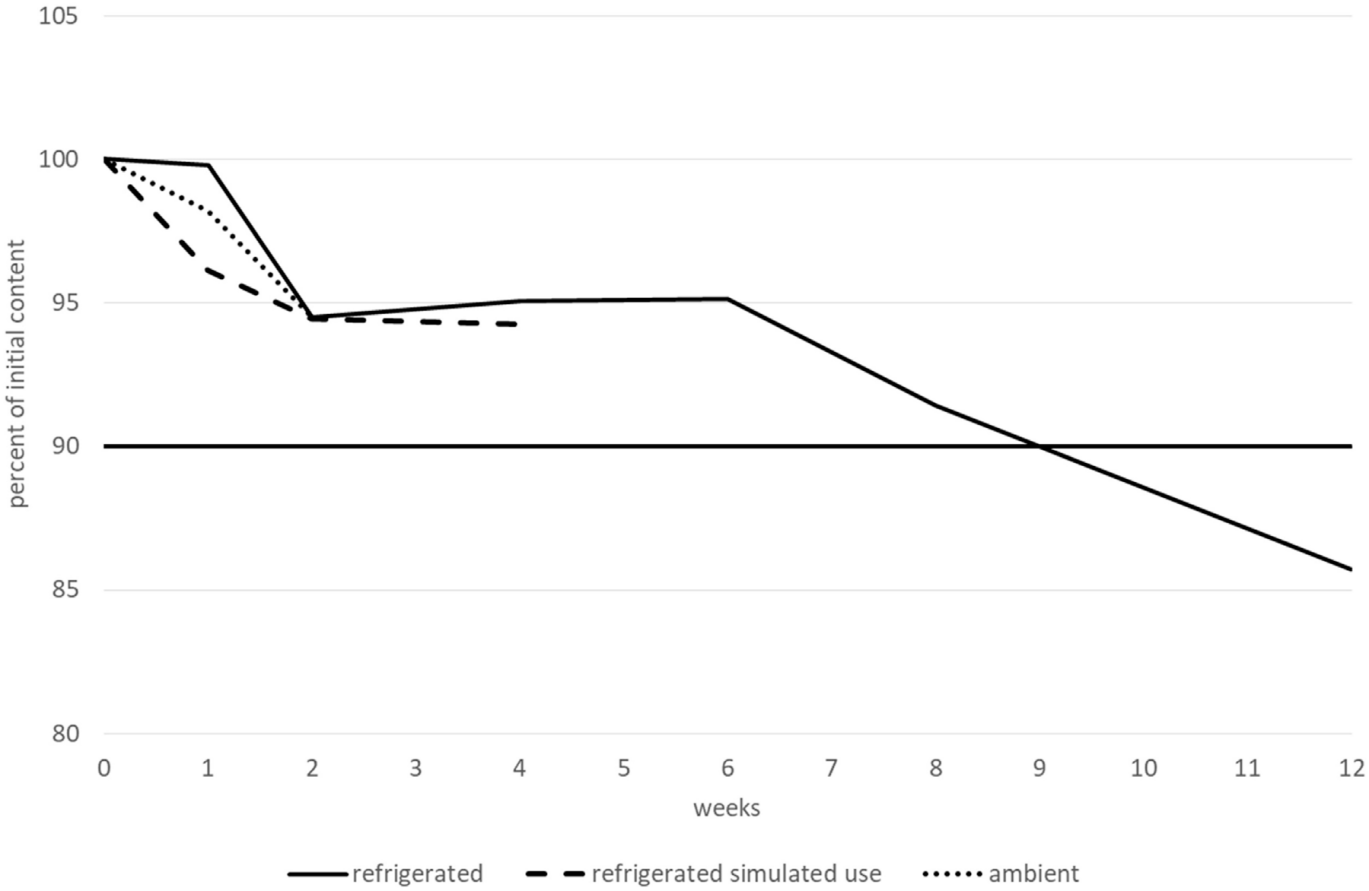
Betaxolol hydrochloride content variations during the stability study.

The betaxolol hydrochloride content remained higher than 90% of its initial value for at least 15 days when stored at ambient temperature, and for at least 1 month after opening and when stored at 2–8 °C. However, before opening, betaxolol hydrochloride content remained higher than 90% of its initial value after 2 months when stored at 2–8 °C, but not after 3 months and decreased by approximately 14%. Moreover, after 3 months, two new peaks appeared that could be related to degradation products. The first peak had an RRT of approximately 1.60–1.70 (close to the RRT observed for a degradation product during forced degradation experiments, under extreme light and heat conditions) and a relative area greater than 0.1% (0.15–0.17%). The second one had an RRT of approximately 1.80–1.90, and a relative area of less than 0.10% (0.07–0.09%). None of the other tested parameters (pH, TAMC and TYMC, and viscosity) varied outside of the conformity limits, irrespective of the storage conditions. Therefore, betaxolol hydrochloride 1 mg.mL^−1^ was considered to be stable for 2 months when stored at 2–8 °C, 1 month after opening at 2–8 °C, and for 15 days when stored at 25 °C/60% RH. The variations in betaxolol content during the stability study are represented in Figure 4. Refrigerated conditions are represented by a continuous line, ambient conditions by a dotted line and refrigerated conditions with simulated use by a dashed line. The results (including CI95 for betaxolol content) are detailed in a table in the Supplementary Material.

**FIGURE 5:**
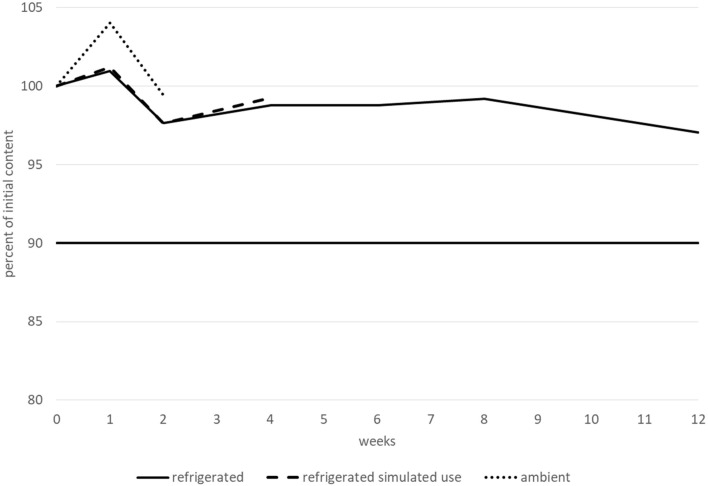
Bisoprolol hemifumarate content variations during the stability study.

The bisoprolol hemifumarate content remained greater than 90% of its initial value for at least 15 days when stored at ambient temperature, for at least 1 month after opening and when stored at 2–8 °C and for 3 months before opening and when stored at 2–8 °C. No degradation products or increases were observed during the entire study. All the other tested parameters (pH, TAMC and TYMC, and viscosity) did not vary outside of the conformity limits, irrespective of the storage conditions, with one notable exception for viscosity when the suspension was stored at 2–8 °C. Under these conditions, the viscosity remained stable for 2 months (with all values between 900 and 997 cP), but dramatically increased after 3 months (to 1,235 cP). Therefore, bisoprolol hemifumarate 0.5 mg.mL^−1^ was considered to be stable for 2 months when stored at 2–8 °C, 1 month after opening at 2–8 °C and for 15 days when stored at 25 °C/60% RH. Variations in bisoprolol content during the stability study are presented in Figure 5. Results (including CI95 for bisoprolol content) are summarised in a table in the Supplementary Material.

**FIGURE 6:**
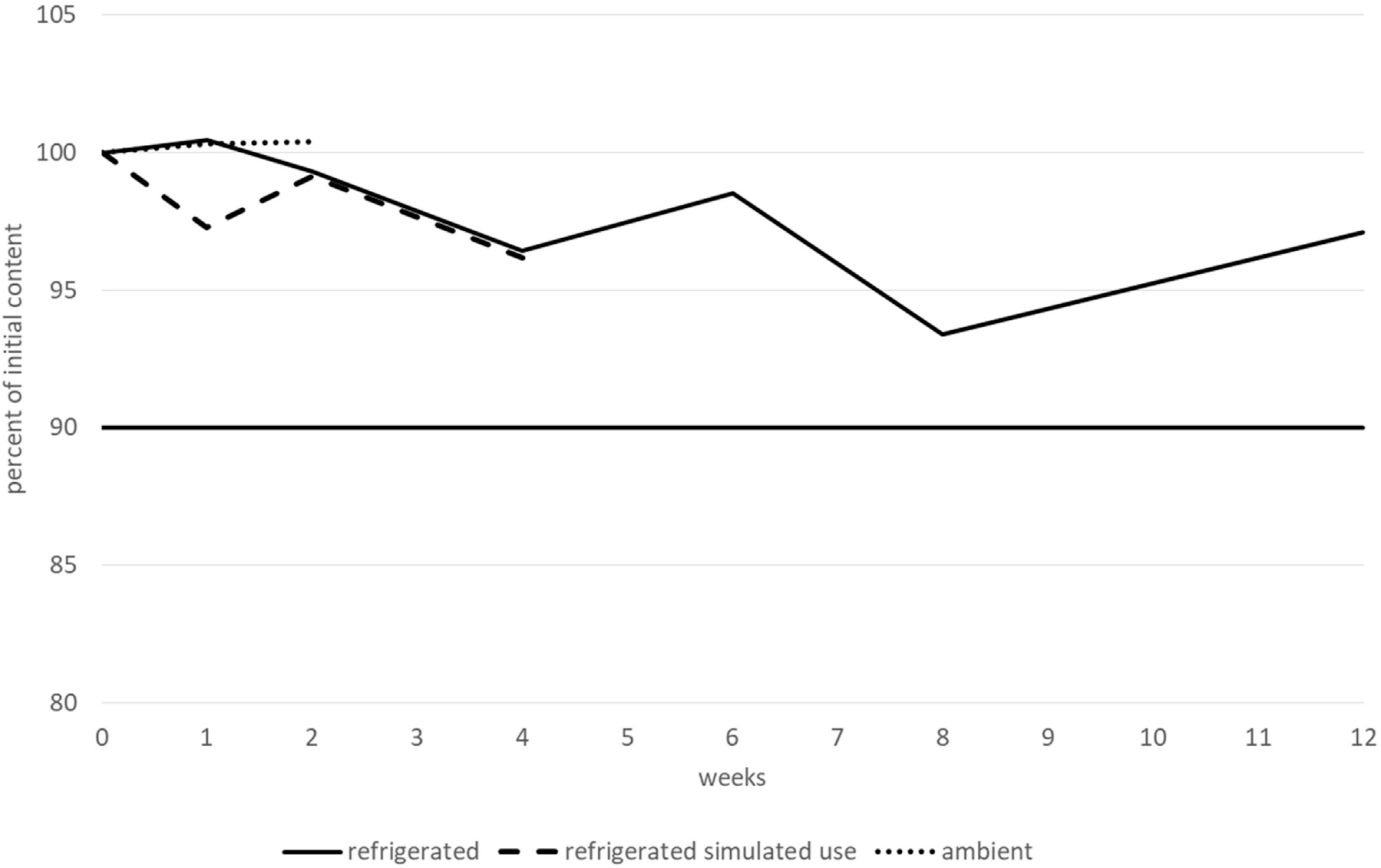
Nadolol content variations during the stability study**.**

During the nadolol stability study, all the tested parameters (nadolol content, pH, TAMC and TYMC, and viscosity) did not vary outside of the conformity limits. Regarding degradation products, only one additional very small peak was observed (RRT 0.26-0.28), but with a relative area remaining lower than 0.10% irrespective of the storage condition. Due to their similar RRTs, this peak could correspond to a degradation product observed during a forced degradation experiment under oxidative conditions. Therefore, nadolol 10 mg.mL^−1^ /potassium sorbate 3 mg.mL^−1^/citric acid 3 mg.mL^−1^ in Syrspend pH 4 can be considered stable for 3 months when stored at 2–8 °C, 1 month after opening at 2–8 °C and for 15 days when stored at 25 °C/60% RH. Variations in nadolol content during the stability study are represented in Figure 6. The results (including CI95 for nadolol content) are summarised in a table in the Supplementary Material.

## Discussion

The dosing methods of betaxolol hydrochloride, bisoprolol hemifumarate, and nadolol were validated as stability-indicating. Forced degradation studies were performed, and proved that our protocol can be used to detect how the stability of the beta-blocker suspensions changes with time. It would have been possible to spike the beta-blockers with a mixture of known degradation products, as it is often done in pharmaceutical companies during method validation, but this was not necessary [24, 25]. The protocol we used to demonstrate the stability-indicating nature of our dosing method is consistent with the vast majority of published stability studies.

In the literature, forced degradation studies have already been performed on beta-blockers with slight differences. For example, betaxolol degradation exceeded 80% under basic conditions (NaOH 5N, 24 h) in two studies, [26, 27], leading to the formation of 1 [26] or 3 [27] degradation products. Bisoprolol forced degradation has also been reported in two studies [28, 29]. The first study [28], in which the authors tried to degrade bisoprolol moderately (by less than 10%), reported some degradation products with RRT and degradation conditions close to those described herein. Finally, nadolol forced degradation has only been described once in the literature [30].

Microbiological methods were also validated. The simplest protocol for routinely determining TAMC and TYMC is the surface spread method. We first tried this protocol without success due to growth inhibition during method validation, probably due to the preservative in Syrspend®. A filtration protocol was then tried, but during our first trials, the membrane clogged. A wide variety of protocols were tried (dilution, prefiltration, other membranes, and so on), but we finally found that membrane clogging was due to starch, one of the main constituents of Syrspend®. Adding α-amylase before filtration allowed starch lysis, enabling easy filtration without causing growth inhibition. We recommend this protocol for TAMC and TYMC determination of drugs compounded with Syrspend®, irrespective of the active pharmaceutical ingredient.

We also found that the preservative in Syrspend®, sodium benzoate, was effective for betaxolol hydrochloride 1 mg.mL^−1^ without the need for any additives. However, the preservative efficacy test failed with nadolol 10 mg.mL^−1^ (for two of the tested strains: *Aspergillus* and *Pseudomonas*). When we measured the pH of nadolol 10 mg.mL^−1^ in Syrspend® without any additives, we found it to be approximately 9.0. At this pH, sodium benzoate is generally not effective [31], so we tried using several buffers to acidify nadolol 10 mg.mL^−1^. The most effective was citric acid 3 mg.mL^−1^, resulting in a pH of 5.5, at which point sodium benzoate is effective. Moreover, citric acid has its own preservative effect [32], is very safe to use [33], and can easily be administered in paediatric practice. However, as the first tests for preservative efficacy were not compliant (for one of the tested strains: *Aspergillus*), we decided to add a second preservative frequently used in paediatric compounding practice, potassium sorbate. Finally, nadolol 10 mg.mL^−1^ with citric acid 3 mg.mL^−1^ and potassium sorbate 3 mg.mL^−1^ in Syrspend® was compliant throughout the entire stability study when preservative efficacy was tested. Surprisingly, bisoprolol hemifumarate 0.5 mg.mL^−1^ also failed the preservative efficacy test, despite having an acidic pH (approximately 4.6). However, the mixture of bisoprolol hemifumarate 0.5 mg.mL^−1^ with citric acid 3 mg.mL^−1^ and potassium sorbate 3 mg.mL^−1^ in Syrspend® passed the preservative efficacy test throughout the entire stability study.

Both bisoprolol hemifumarate 0.5 mg.mL^−1^ and nadolol 10 mg.mL^−1^ contain a mixture of preservatives: potassium sorbate 3 mg.mL^−1^, citric acid 3 mg.mL^−1^ and less than 1 mg.mL^−1^ of sodium benzoate. A number of studies have argued that citric acid could interact with sodium benzoate based on similarities between ascorbic and citric acids, despite there being no proof or scientific report of this. There are scientific reports on the interaction between sodium benzoate and ascorbic acid in beverages, which can result in the formation of carcinogenic benzene [34]. Exposure to ultraviolet (UV) light and elevated temperatures during storage can lead to benzene formation in products containing benzoic acid and ascorbic acid [35]. A large-scale study conducted on beverages yielded the reformulation of several of them (with the suppression of sodium benzoate or ascorbic acid) [36], but no correlation was found between the presence of citric acid/sodium benzoate and benzene formation despite the high prevalence of these substances in beverages. Moreover, another study showed that the mixture of ascorbic acid, sodium benzoate, citric acid and Cu^2+^ could favour benzene formation *in vitro*; however, benzene was not found in a mixture with citrate buffer, sodium benzoate, and Cu^2+^ without ascorbic acid [37]. Therefore, we can conclude that the mixture of “citric acid 3 mg.mL^−1^ and less than 1 mg.mL^−1^ of sodium benzoate” can be used as a preservative for bisoprolol hemifumarate and nadolol suspensions without any risk of benzene formation under normal storage conditions.

The safety of the preservatives used in paediatric practice is a topical issue. A recent work analysed the composition of 219 oral liquid formulations prescribed in paediatric and neonatology units, listed the most commonly used excipients and reviewed their toxicity data [38]. The most commonly used antimicrobial preservatives were parabens (in 82% of oral medications), citric acid (also used as a buffering agent, in 36% of oral medications) and sodium benzoate (in 26% of oral medications). Although the toxicity of parabens is still being discussed [39], their prevalence in commercialised drugs and their bioaccumulation [40, 41] should make them an ingredient to be avoided in compounded paediatric formulations. Conversely, neonates appear to lack the capacity to conjugate sodium benzoate with glycine, [41, 42] which can cause metabolic acidosis and neurotoxicity due to benzoic acid. However, sodium benzoate has been found in several drugs administered to neonates, and the EMA recommends a maximum dose of 5 mg.kg^−1^ per day, not just paediatric patients [38]. Syrspend® pH 4 contains less than 1 mg.mL^−1^ (1%) of sodium benzoate. Finally, there are no data showing any risk with potassium sorbate used as a preservative if its daily intake remains lower than 3 mg.kg^−1^ per day [43].

In our country, the regulatory authority requires pharmacists to compound a formula using pharmaceutical-grade raw material wherever possible, as detailed in the Good Compounding Practices [44]. Here, nadolol raw materials were available and were therefore used for the nadolol 10 mg.mL^−1^ oral suspension. However, our attempts to obtain betaxolol and bisoprolol raw materials packaged in bulk and suitable for hospital preparation were unsuccessful, and we were forced to use tablets for betaxolol 1 mg.mL^−1^ and bisoprolol hemifumarate 0.5 mg.mL^−1^. This is one of the limitations of our study, as the excipients in the tablets could have influenced the stability or the quality of the formulations.

The stability of betaxolol compounded as a 1 mg.mL^−1^ oral suspension has only been studied once [45], in water, Inorpha®, and Ora® mixtures, and it was reported to be no less than 14 days, irrespective of the excipient or temperature (4 °C or 20 °C); however, the authors did not study stability beyond 14 days. The stability of bisoprolol compounded for oral administration has not been identified in the literature. Finally, nadolol 10 mg.mL^−1^ oral suspensions were reported in two publications to be stable for at least 90 days, in Ora® mixtures [46] and in Syrspend® [47], but these studies did not evaluate the efficacy of the preservatives, and the tested formulas did not contain citric acid and potassium sorbate to improve this parameter.

## Conclusion

Betaxolol hydrochloride 1 mg.mL^−1^ in Syrspend® pH 4 can be compounded and stored for 2 months at 2–8 °C, 1 month after opening at 2–8 °C and for 15 days at ambient temperature. Bisoprolol hemifumarate 0.5 mg.mL^−1^ and nadolol 10 mg.mL^−1^ in Syrspend® pH 4 can also be compounded, but require the addition of a buffer and a preservative (citric acid 3 mg.mL^−1^ and potassium sorbate 3 mg.mL^−1^) for storage. With these additions, bisoprolol hemifumarate 0.5 mg.mL^−1^ is stable for 2 months when stored at 2–8 °C, 1 month after opening at 2–8 °C and for 15 days when stored at 25 °C/60% RH, and nadolol 10 mg.mL^−1^ is stable for 3 months when stored at 2–8 °C, 1 month after opening at 2–8 °C, and for 15 days when stored at 25 °C/60% RH.

These beta-blocker liquid oral formulations can be routinely compounded and used in paediatric care practice. Moreover, they can be useful as therapeutic alternatives if one of these beta-blockers is in short supply.

## Data Availability

The original contributions presented in the study are included in the article/Supplementary Material, further inquiries can be directed to the corresponding author.
