# Supplementary material for: Compounding and stability studies of liquid oral formulations of beta-blockers (bisoprolol, betaxolol, and nadolol) for paediatric patients
Source: J Pharm Pharm Sci. 2025 Dec 2;28:15387. doi: 10.3389/jpps.2025.15387 (PMC12705468; doi:10.3389/jpps.2025.15387)
Supplement: Supplementary file 1 [file DataSheet4.docx]

**Table S1. Betaxolol chlorhydrate stability study**

| Storage condition | Parameter | D0 | D7 | D15 | D30 | D45 | D60 | D90 |
| --- | --- | --- | --- | --- | --- | --- | --- | --- |
| +2 ^o^C / +8 ^o^C | Content +/- CI95 (mg.mL^-1^) | 0.958 +/- 0.031 | 0.956 +/- 0.011 | 0.905 +/- 0.017 | 0.910 +/- 0.012 | 0.911 +/- 0.010 | 0.875 +/- 0.012 | 0.821 +/- 0.031 |
|  | pH | 4.47 | 4.38 | 4.43 | 4.46 | 4.43 | 4.39 | 4.36 |
|  | TAMC/TYMC | C | C | C | C | C | C | C |
|  | Preservative efficacy | C | *NR* | *NR* | C | *NR* | C | C |
|  | Viscosity (cP) | 484 | 490 | 509 | 490 | 454 | 440 | 439 |
| +2 ^o^C / +8 ^o^C, simulated use | Content +/- CI95 (mg.mL^-1^) | 0.958 +/- 0.031 | 0.921 +/- 0.010 | 0.904 +/- 0.009 | 0.902 +/- 0.009 | *NR* | *NR* | *NR* |
|  | pH | 4.47 | 4.38 | 4.41 | 4.42 | *NR* | *NR* | *NR* |
|  | TAMC/TYMC | C | C | C | C | *NR* | *NR* | *NR* |
|  | Preservative efficacy | C | *NR* | *NR* | C | *NR* | *NR* | *NR* |
| +25 ^o^C / 60% RH | Content +/- CI95 (mg.mL^-1^) | 0.958 +/- 0.031 | 0.940 +/- 0.013 | 0.905 +/- 0.021 | *NR* | *NR* | *NR* | *NR* |
|  | pH | 4.47 | 4.40 | 4.38 | *NR* | *NR* | *NR* | *NR* |
|  | TAMC/TYMC | C | C | C | *NR* | *NR* | *NR* | *NR* |
|  | Preservative efficacy | C | *NR* | C | *NR* | *NR* | *NR* | *NR* |
|  | Viscosity (cP) | 484 | 527 | 530 | *NR* | *NR* | *NR* | *NR* |

*NR*: analysis not realized

**Table S2. Bisoprolol hemifumarate stability study**

| Storage condition | Parameter | D0 | D7 | D15 | D30 | D45 | D60 | D90 |
| --- | --- | --- | --- | --- | --- | --- | --- | --- |
| +2 ^o^C / +8 ^o^C | Content +/- CI95 (mg.mL^-1^) | 0.473 +/- 0.027 | 0.478 +/- 0.014 | 0.462 +/- 0.010 | 0.467 +/- 0.015 | 0.467 +/- 0.024 | 0.469 +/- 0.017 | 0.459 +/- 0.012 |
|  | pH | 4.38 | 4.58 | 4.67 | 4.61 | 4.46 | 4.53 | 4.36 |
|  | TAMC/TYMC | C | C | C | C | C | C | C |
|  | Preservative efficacy | C | *NR* | *NR* | C | *NR* | C | C |
|  | Viscosity (cP) | 979 | 921 | 901 | 900 | 997 | 952 | 1235 |
| +2 ^o^C / +8 ^o^C, simulated use | Content +/- CI95 (mg.mL^-1^) | 0.473 +/- 0.027 | 0.479 +/- 0.012 | 0.462 +/- 0.009 | 0.469 +/- 0.007 | *NR* | *NR* | *NR* |
|  | pH | 4.38 | 4.58 | 4.61 | 4.61 | *NR* | *NR* | *NR* |
|  | TAMC/TYMC | C | C | C | C | *NR* | *NR* | *NR* |
|  | Preservative efficacy | C | *NR* | *NR* | C | *NR* | *NR* | *NR* |
| +25 ^o^C / 60% RH | Content +/- CI95 (mg.mL^-1^) | 0.473 +/- 0.027 | 0.492 +/- 0.026 | 0.470 +/- 0.012 | *NR* | *NR* | *NR* | *NR* |
|  | pH | 4.38 | 4.37 | 4.69 | *NR* | *NR* | *NR* | *NR* |
|  | TAMC/TYMC | C | C | C | *NR* | *NR* | *NR* | *NR* |
|  | Preservative efficacy | C | *NR* | C | *NR* | *NR* | *NR* | *NR* |
|  | Viscosity (cP) | 979 | 905 | 898 | *NR* | *NR* | *NR* | *NR* |

*NR*: analysis not realized

**Table S3. Nadolol stability study**

| Storage condition | Parameter | D0 | D7 | D15 | D30 | D45 | D60 | D90 |
| --- | --- | --- | --- | --- | --- | --- | --- | --- |
| +2 ^o^C / +8 ^o^C | Content +/- CI95 (mg.mL^-1^) | 10.94 +/- 0.32 | 10.99 +/- 0.19 | 10.87 +/- 0.24 | 10.55 +/- 0.39 | 10.78 +/- 0.41 | 10.22 +/- 0.29 | 10.62 +/- 0.23 |
|  | pH | 5.16 | 4.95 | 4.97 | 5.00 | 5.04 | 5.01 | 5.02 |
|  | TAMC/TYMC | C | C | C | C | C | C | C |
|  | Preservative efficacy | C | *NR* | *NR* | C | *NR* | C | C |
|  | Viscosity (cP) | 623 | 606 | 644 | 587 | 559 | 623 | 602 |
| +2 ^o^C / +8 ^o^C, simulated use | Content +/- CI95 (mg.mL^-1^) | 10.94 +/- 0.32 | 10.64 +/- 0.70 | 10.85 +/- 0.29 | 10.52 +/- 0.22 | *NR* | *NR* | *NR* |
|  | pH | 5.16 | 4.95 | 4.93 | 5.02 | *NR* | *NR* | *NR* |
|  | TAMC/TYMC | C | C | C | C | *NR* | *NR* | *NR* |
|  | Preservative efficacy | C | *NR* | *NR* | C | *NR* | *NR* | *NR* |
| +25 ^o^C / 60% RH | Content +/- CI95 (mg.mL^-1^) | 10.94 +/- 0.32 | 10.97 +/- 0.20 | 10.98 +/- 0.37 | *NR* | *NR* | *NR* | *NR* |
|  | pH | 5.16 | 4.98 | 4.98 | *NR* | *NR* | *NR* | *NR* |
|  | TAMC/TYMC | C | C | C | *NR* | *NR* | *NR* | *NR* |
|  | Preservative efficacy | C | *NR* | C | *NR* | *NR* | *NR* | *NR* |
|  | Viscosity (cP) | 633 | 636 | 667 | *NR* | *NR* | *NR* | *NR* |

*NR*: analysis not realized
