# Supplementary material for: Compounding and stability studies of liquid oral formulations of beta-blockers (bisoprolol, betaxolol, and nadolol) for paediatric patients
Source: J Pharm Pharm Sci. 2025 Dec 2;28:15387. doi: 10.3389/jpps.2025.15387 (PMC12705468; doi:10.3389/jpps.2025.15387)
Supplement: Supplementary file 2 [file DataSheet3.docx]

**Nadolol forced degradation chromatograms**

**Figure S1. Light (sunlamp, 14 d)**

**Figure S2. Heat (80^o^C, 14 d)**

**Figure S3. Acidic (HCl 2.5 M, 14 d)**

**Figure S4. Alkaline (NaOH 4 M, 6 d)**

**Figure S5. Oxidation (H_2_O_2_ 15%, 5 h)**
