# Supplementary material for: Compounding and stability studies of liquid oral formulations of beta-blockers (bisoprolol, betaxolol, and nadolol) for paediatric patients
Source: J Pharm Pharm Sci. 2025 Dec 2;28:15387. doi: 10.3389/jpps.2025.15387 (PMC12705468; doi:10.3389/jpps.2025.15387)
Supplement: Supplementary file 3 [file DataSheet2.docx]

**Bisoprolol hemifumarate forced degradation chromatograms**

**Figure S1. Light (sunlamp, 2 d)**

**Figure S2. Heat (50^o^C, 2 d)**

**Figure S3. Acidic (HCl 2M, 1 h)**

**Figure S4. Alkaline (NaOH 0.1 M, 15 min)**

**Figure S5. Oxidation (H_2_O_2_ 3%, 15 min)**
