# Supplementary material for: Compounding and stability studies of liquid oral formulations of beta-blockers (bisoprolol, betaxolol, and nadolol) for paediatric patients
Source: J Pharm Pharm Sci. 2025 Dec 2;28:15387. doi: 10.3389/jpps.2025.15387 (PMC12705468; doi:10.3389/jpps.2025.15387)
Supplement: Supplementary file 4 [file DataSheet1.docx]

**Betaxolol chlorhydrate forced degradation chromatograms**

**Figure S1. Light (sunlamp, 7 d)**

**Figure S2. Heat (80^o^C, 5 d)**

**Figure S3. Acidic (HCl, 2.5M, 15 min)**

**Figure S4. Alkaline (NaOH 0.1 M, 30 min)**

**Figure S5. Oxidation (H_2_O_2_ 3%, 2 h)**
